# Assays for Identification and Differentiation of *Brucella* Species: A Review

**DOI:** 10.3390/microorganisms10081584

**Published:** 2022-08-06

**Authors:** Berzhan Kurmanov, Diansy Zincke, Wanwen Su, Ted L. Hadfield, Alim Aikimbayev, Talgat Karibayev, Maxat Berdikulov, Mukhit Orynbayev, Mikeljon P. Nikolich, Jason K. Blackburn

**Affiliations:** 1 Spatial Epidemiology & Ecology Research Lab, Department of Geography, University of Florida, Gainesville, FL 32611, USA; 2 Emerging Pathogens Institute, University of Florida, Gainesville, FL 32611, USA; 3 Bacterial Diseases Branch, Walter Reed Army Institute of Research, Silver Spring, MD 20910, USA; 4 Scientific Practical Center for Sanitary Epidemiological Expertise and Monitoring, Ministry of Health, Almaty 050008, Kazakhstan; 5 National Reference Veterinary Center, Nur-Sultan 010000, Kazakhstan; 6 Research Institute for Biological Special Problems, Otar, Zhambyl 080409, Kazakhstan

**Keywords:** *Brucella abortus*, *Brucella canis*, *Brucella melitensis*, *Brucella suis*, species identification, molecular techniques

## Abstract

Brucellosis is one of the most important and widespread bacterial zoonoses worldwide. Cases are reported annually across the range of known infectious species of the genus *Brucella.* Globally, *Brucella melitensis*, primarily hosted by domestic sheep and goats, affects large proportions of livestock herds, and frequently spills over into humans. While some species, such as *Brucella abortus*, are well controlled in livestock in areas of North America, the Greater Yellowstone Ecosystem supports the species in native wild ungulates with occasional spillover to livestock. Elsewhere in North America, other *Brucella* species still infect domestic dogs and feral swine, with some associated human cases. *Brucella* spp. patterns vary across space globally with *B. abortus* and *B. melitensis* the most important for livestock control. A myriad of other species within the genus infect a wide range of marine mammals, wildlife, rodents, and even frogs. Infection in humans from these others varies with geography and bacterial species. Control in humans is primarily achieved through livestock vaccination and culling and requires accurate and rapid species confirmation; vaccination is *Brucella* spp.-specific and typically targets single livestock species for distribution. Traditional bacteriology methods are slow (some media can take up to 21 days for bacterial growth) and often lack the specificity of molecular techniques. Here, we summarize the molecular techniques for confirming and identifying specific *Brucella* species and provide recommendations for selecting the appropriate methods based on need, sensitivity, and laboratory capabilities/technology. As vaccination/culling approaches are costly and logistically challenging, proper diagnostics and species identification are critical tools for targeting surveillance and control.

## 1. Introduction

Brucellosis, also known as “Malta fever” or “undulant fever”, is an important zoonotic infection, affecting both domestic and wild animals and humans. The host range of the infection includes ungulates, carnivores, rodents, primates, and marine mammals. In unvaccinated animals, brucellosis causes various disorders such as abortion, placentitis, orchitis, epididymitis, etc. [1]. This often results in heavy economic losses associated with a decrease in reproductive efficiency of the livestock and animal replacement costs associated with herd culling [2,3]. Human infections are associated with direct contact with infected animals or their products; by ingestion of contaminated dairy products, primarily cheese and unpasteurized milk; and exposure to infectious aerosols. Human brucellosis is rarely fatal, but dramatically affects various body systems (reproductive, musculoskeletal, central nervous, etc.) and can cause severe and sometimes lasting sequalae including disabilities [4,5]. Several live-attenuated vaccines were developed using attenuated mutant *Brucella* strains, such as *B. melitensis* Rev.1, *B. abortus* S19, and *B. abortus* RB51 [6]. However, despite the effectiveness of these vaccines for livestock immunization, they proved unsafe for humans. This fact, along with the difficulties in providing adequate antimicrobial treatment, makes control of animal brucellosis the most important component for the prevention of human disease [7]. In the United States, human brucellosis rarely occurs because of an effective animal disease control program for cattle [8]; cases of *B. suis* [9] and *B. canis* [10] continue. However, in many brucellosis-endemic countries, even those applying livestock vaccination, the infection still represents a significant and uncontrolled problem [11].

The etiologic agents of brucellosis are small Gram-negative aerobic coccobacilli belonging to the genus *Brucella*. The bacteria are characterized as facultative intracellular parasites lacking flagellae, capsules, endospores, and plasmids [12] and replicating within monocyte–macrophage cells [13]. The identification of *Brucella* species is based on phenotypic traits including biochemical testing and serological assays. Phenotypically, *Brucella* species can be divided into “smooth” (S-type) and “rough” (R-type) depending on the structure of their lipopolysaccharide (LPS), the major component of the outer membrane of Gram-negative bacteria. The LPS of S-type *Brucella* species has the O-sidechain (also called O-polysaccharide or O antigen), providing protection from lytic agents produced by eukaryotic host cells, and protecting infected host cells from apoptosis. The R-type species do not produce the O-chain, and, thus, can be eliminated more readily by the host immune system [14].

Until the middle of the 1990s, the genus *Brucella* consisted of six “classical” or “core” species: *Brucella abortus*, *Brucella canis*, *Brucella melitensis*, *Brucella neotomae*, *Brucella ovis*, and *Brucella suis*. Then, several new (nonclassical) strains were isolated from marine mammals and assigned to a novel species designated as *Brucella maris*, which was later split into two separate species: *Brucella ceti* (circulating in cetaceans: whales, dolphins, and porpoises) and *Brucella pinnipedialis* (mainly infecting seals) [15]. Since 2008, four additional species have been reported: *Brucella inopinata*, *Brucella microti*, *Brucella papionis*, and *Brucella vulpis* [16]. The data on host specificity and serotypes for the 12 currently known species [17,18,19] are shown in Table 1. Recently, several atypical *Brucella*-like strains were isolated from Australian rodents [20], bullfrogs [21], and a bluespotted ribbontail ray [22].

Three S-type *Brucella* species, *B. melitensis*, *B. abortus*, and *B. suis*, are responsible for the majority of livestock and human brucellosis [23] cases. In modern *Brucella* systematics, these species are divided into 15 biovars (bv; biotypes): *B. melitensis* bv. 1–3, *B. abortus* bv. 1–6 and 9, and *B. suis* bv.1–5. The *B. abortus* bv 7 and bv 8 were removed from the *Brucella* nomenclature in 1986 and 1978, respectively [24]. Five *Brucella* species, *B. canis*, *B. ceti*, *B. inopinata*, *B. pinnipedialis*, and *B. neotomae* [18,25,26,27], as well as the bv 2 of *B. suis*, are hypothesized to be associated with more moderate forms of brucellosis in humans. No cases of human brucellosis caused by *B. ovis*, *B. microti*, *B. papionis*, *B. vulpis*, and biovar 5 of *B. suis* have been reported [28,29].

The traditional system of *Brucella* diagnosis includes clinical examination, cultivation of bacterial isolates from biological samples (i.e., blood, tissue, cerebrospinal fluid, and urine), microscopy, biochemical tests (fermentation tests, catalase, oxidase, and urease), and serological tests (Rose Bengal test, serum/latex agglutination test, complement fixation test, and enzyme-linked immunosorbent assay) [30,31,32]. These methods are generally time-consuming (may take days for detectable growth); some methods have low sensitivity and/or specificity, and require laboratory-qualified personnel, as well as a biosafety level 3 facility [33,34]. Molecular biology techniques have improved the identification of *Brucella* from cultures but are less successful for the speciation of *Brucella*. Discrimination of *Brucella* spp. at the genus level using PCR-based techniques is usually straightforward, while the interspecific differentiation is much more complicated because of the *high degree of genome sequence similarity* between different *Brucella* species (greater than 95%) [35]. As demonstrated recently, a case of human brucellosis was caused by an amphibian-type *Brucella inopinata-*like strain. The strain could not be characterized classically or diagnosed serologically, with molecular tools essential to diagnosis [36].

Many of the published *Brucella* PCR assays are based on polymorphisms arising from species-specific localization of an 842 bp mobile genetic element IS*711*, also known as IS*6501*, which is characteristic for the *Brucella* genus and not shared by the near-neighbor genus *Ochrobactrum*. The pattern of IS*711* distribution in the genomes of different *Brucella* species is relatively unique and stable. The copy number of IS*711* ranges from 7 in *B. abortus*, *B. melitensis*, and *B. suis* to more than 30 in *B. ceti*, *B. ovis*, and *B. pinnipedialis* [37,38]. Another chief source of *Brucella* genomic diversity are insertions and deletions of nucleotide sequences (‘indels’) in various genes, ranging from a dozen to thousands of nucleotides [39,40]. The IS*711* mobile element and indel polymorphisms can be easily detected using PCR methods.

## 2. Genotyping

Genotyping of *Brucella* spp. mostly relies on other types of genetic markers: restriction fragment length polymorphisms (RFLP), tandem repeats (TRs), and single nucleotide polymorphisms (SNPs) which tend to cluster isolates by phenotypically identified species. RFLP patterns are the result of restriction-digested DNA from a pure sample or an amplified gene. The restriction fragments are electrophoresed to generate a ladder of fragment sizes from large (top) to small (bottom) on a gel or membrane. The resulting pattern is unique for each restriction enzyme. Analysis is best accomplished using computer software specific for the RFLP genetic analysis. This method generally can genotype isolates and differentiate between closely related species.

A tandem repeat is defined as a series of end-to-end duplications of a core DNA sequence, ranging from 2 to 60 (and more) nucleotides. Tandem repeats (also known as simple sequence repeat (SSR), short tandem repeat (STR), simple sequence length polymorphism (SSLP), and variable-number tandem repeat (VNTR)) are widely distributed throughout the genomes of most eukaryotic and prokaryotic organisms. The number of repeats in a particular TR locus can vary between species and strains, allowing their typing by conventional PCR or MLVA assays [41,42,43]. Single-nucleotide polymorphisms (SNPs) are the most common type of genetic variations in the genomes of all living creatures. Whole-genome analysis of *Brucella* spp. revealed hundreds of SNPs, allowing discrimination not only of species, but also biovars [44,45,46], although phenotypic biovars may be scattered among genotype clusters. Until recently, SNP typing was a more difficult task than the identification of other types of polymorphisms, such as indel or TR, and, hence, required more advanced techniques: Melt-MAMA assay, real-time assays, restriction fragment length polymorphism analysis (RFLP), TaqMan allelic discrimination, high-resolution melt analysis (HRMA), or DNA sequencing.

Here, we review the main approaches in PCR-based techniques for the identification and differentiation of *Brucella* species. The nucleotide sequences of the target loci, primers, and probes mentioned in the reviewed publications were checked using the BLAST (Basic Local Alignment Search Tool, https://blast.ncbi.nlm.nih.gov/Blast.cgi, accessed on 1 September 2019) queries of the GenBank sequence database (https://www.ncbi.nlm.nih.gov/genbank, accessed on 1 September 2019) kept by the National Center for Biotechnology Information (NCBI), and all identified discrepancies were indicated. The names of genes and loci are either given as were indicated in the original publications, or we use the names of analogue loci from known reference strains, such as *Brucella abortus* bv. 2 str. ATCC 23449 (taxid:520450), *Brucella melitensis* ATCC 23456 (taxid:224914), and others. Most authors of the publications reviewed herein tested the effectiveness of their assays using six classical *Brucella* species: *B. abortus* biovars 1–6 and 9, *B. melitensis* biovars 1–3, *B. suis* biovars 1–5, *B. canis*, *B. ovis*, and *B. neotomae.* To avoid listing these each time, we, henceforth, refer to this list as the “core panel”.

## 3. Conventional PCR

Conventional PCR applies DNA amplification with endpoint analysis of products using gel electrophoresis. This technique is relatively simple and cost-effective. Conventional PCR assays can be designed to detect one (uniplex PCR) or several (multiplex PCR) targets in a one-test format. Uniplex and duplex PCR assays are the most common varieties of PCR used for *Brucella* identification [47,48,49,50,51,52]. For the most part, these simpler PCR approaches are not very efficient as they are able to establish the affiliation only to the genus or, at best, to one of the species, and are unable to discriminate the main *Brucella* species. The most common targets for such assays are the sequences encoding 16S and 23S rRNAs, BCSP31 protein, and IS*711* transposase. Multiplex PCR assays that employ primers for more than two targets allow for the identification of a range of *Brucella* species in a single reaction tube. Therefore, their discriminatory power is significantly higher than single-target assays. The most well-known and broadly used multiplex conventional PCR assays for *Brucella* are the AMOS and Bruce-ladder assays, including their various modifications.

The AMOS PCR assay was designed by Bricker and Halling [53] for the discrimination of four *Brucella* species: *B. abortus*, *B. melitensis*, *B. ovis*, and *B. suis* (AMOS). The original assay was composed of five oligonucleotide primers: four forward (each unique for one of the four target species) and one universal reverse (located within IS*711*) (Table S1, index A1). The assay was tested on 20 strains of the *Brucella* core panel and 8 strains of a non-*Brucella* panel (Table 2, genera # 6, 11, 28, 45, 56, 58, and 63). The specific products were amplified only in four *Brucella* species, and within these species, only in certain biovars: *B. abortus* bv. 1, 2, and 4 (498 bp); *B. melitensis* bv. 1–3 (731 bp); *B. suis* bv. 1 (285 bp); and *B. ovis* (976 bp) (Table S2, index A1). Despite the incomplete species specificity of the assay, it was considered sufficient for identification in the US, since these biovars were typically being isolated from local livestock. The sensitivity of the AMOS PCR assay was about 7 × 10^3^ bacterial cells (*B. abortus*). The non-*Brucella* panel tested negative. In general, the assay proved to be simple and effective with the limitation that it could not detect all biovars of the four target *Brucella* species, nor discriminate between vaccine and field strains. The same authors, Bricker and Halling [54], in 1995 proposed to add three new primers to the AMOS PCR assay to allow differentiation of the *B. abortus* vaccine strains RB51 and S19 (Table S1, index A2). The RB51/2308 primer paired with the universal reverse primer of the AMOS assay amplified a 364 bp DNA fragment located at the junction of two adjacent IS*711* elements, characteristic of the vaccine strain RB51 and its virulent parent strain, *B. abortus* 2308. However, this fragment is also produced by *B. ovis* strains, which indicates the presence of the same tandemly arranged IS*711* copies in their genomes. The third set of primers (*eri* primer pair) amplified a 178 bp fragment within the *eryC*–*eryD* genes in all *Brucella* species except S19, which had a 702 bp deletion in this region. The combination of these new primers with the original AMOS PCR assay produced unique electrophoresis band profiles of the *B. abortus* vaccine strains RB51 and S19, allowing their reliable differentiation from other *Brucella* strains (Table S2, index A2).

Ewalt and Bricker [55] later reported a new assay called BaSS-PCR (*Brucella abortus* species-specific), comprising primers for identification of four *B. abortus* genome targets: the *B. abortus* specific primers from the original AMOS assay, *RB51-3* primer to identify the RB51 vaccine strain, *Ery* primer set to identify the S19 vaccine strain, and 16S rRNA primers as an internal control (Tables S1 and S2, index A3). In another study, the assay was tested in two separate laboratories using 60 strains of the *Brucella* core panel, 38 *B. abortus* vaccine strains (S19 and RB51), 168 *B. abortus* field isolates, and 68 non-*Brucella* strains (Table 2, genera # 9, 28, 34, 36, 39, 49, 53, 60, 64, and 66) [56]. The specificity of the assay was 93.2–99.7% for laboratory 1 and 100% for the laboratory 2. The sensitivity of the assay was 66.7–100% and 100%, respectively. The authors hypothesized the reduced levels of specificity and sensitivity showed by laboratory 1 were associated with a cross-contamination during sample preparation. The assays accurately identified RB51 and distinguished it from the parental strain *B. abortus* 2308.

Ocampo-Sosa et al. [57] described another modification of the AMOS PCR (Table S1, index A4). They redesigned *Eri* primers for the above-mentioned *eryC*–*eryD* region to obtain two variants of the product: a 1269 bp fragment, specific for all *Brucella* strains except for the *B. abortus* S19 vaccine strain, and a 564 bp fragment, specific only for strain S19. In addition, they introduced a new DEL569 primer that, in combination with the universal IS*711* reverse primer of the AMOS assay, should produce a 1740 bp fragment specific for *B. abortus* biovars 5, 6, 9, as well as a subgroup 3b of biovar 3. The enhanced AMOS-ERY assay was tested on 20 isolates representing the core *Brucella* species excluding *B. neotomae*, and 135 field isolates (belonging to *B. abortus*, *B. melitensis*, *B. ovis*, *B. ceti*, and *B. pinnipedialis*). All seven non-*Brucella* strains used in this study (Table 2, genera # 6, 38, 56, and 63) tested negative. The *Brucella* strains were divided into seven groups (genotypes) designated by Latin letters from A to G. As expected, *B. abortus* strains of biovars 5, 6, 9, and 3b shared the same PCR pattern (group D), while vaccine strain S19 was assigned to a unique group A (Table S2, index A4). However, vaccine strains RB51 and Rev1 could not be discriminated by this assay.

Huber et al. [58] expanded the standard AMOS assay with seven new primer sets (*B_mic_spec*, *B_neo*, *B_ov_pin*, *B_ab*, *B_can*, *B_su134*, and *B_su25*) and a single primer 26A (Table S1, index A5). The primers were designed to detect indel polymorphisms specific for certain *Brucella* species. The resulting assay was tested on 131 pure culture and field isolates of *Brucella* spp. (the core panel, *B. ceti*, *B. pinnipedialis*, and *B. microti*), as well as on 18 non-*Brucella* strains (Table 2, genera # 28, 29, 45, and 70). According to the authors, the assay was able to differentiate 9 of the 12 known *Brucella* species (Table S2, index A5). No amplification products were produced from the non-*Brucella* strains tested. The low analytical sensitivity and specificity of the assay (about 5 ng DNA in a 25 μL reaction volume) suggest its usefulness may be limited.

García-Yoldi et al. [59] described a multiplex PCR assay called Bruce-ladder, comprising eight primer pairs (Table S1, index A6) for the identification and differentiation of the most *Brucella* species including the vaccine strains S19, RB51, and Rev.1. The discrimination between *Brucella* species was based on genetic polymorphisms arising from various types of mutations: insertions, deletions, and a single nucleotide substitution. The Bruce-ladder assay was tested on 72 *Brucella* reference strains and isolates (the core panel, *B. pinnipedialis*, *B. ceti*, and the vaccine strains). The specificity of the assay was tested against an extended panel of 23 non-*Brucella* strains (Table 2, genera # 2, 6, 10, 12, 19, 22, 28, 32, 45, 49, 50, 56, 60, 63, 64, and 66). Using the Bruce-ladder assay, each *Brucella* species and each vaccine strain had its own unique set of PCR products (Table S2, index A6). The only exceptions were *B. ceti* and *B. pinnipedialis* that shared the same PCR pattern. All the non-*Brucella* strains tested negative, thereby demonstrating the specificity of the assay. Despite the clear advantages of Bruce-ladder, it was unable to differentiate *B. microti*, *B. inopinata*, marine mammal *Brucella* species, and some *B. suis*/*B. canis* strains. The Bruce-ladder assay demonstrated high efficiency at low cost, and the World Organization for Animal Health (OIE, Office International des Epizooties) recommended it as a rapid and simple one-step molecular test for the identification and typing of *Brucella* species [60].

López-Goñi et al. [61] reported a new tetraplex PCR assay, “Suis-ladder” (Table S1, index A7), to supplement Bruce-ladder for the accurate identification of *B. suis* at the biovar level and the differentiation of closely related and often confused *B. suis*, *B. canis*, and *B. microti.* The assay was tested on 105 *B. suis* and 18 *B. canis* reference strains and field isolates, as well as 1 isolate of *B. microti.* PCR analysis revealed unique PCR patterns *for B. canis*, *B. microti*, and each *B. suis* biovar (Table S2, index A7). The Suis-ladder was able to correctly identify six *B. canis* strains previously misidentified by Bruce-ladder as *B. suis*. At the same time, Suis-ladder identified several *B. suis* field isolates as belonging to biovar 1, although by classic tests, they were previously assigned to biovar 3.

Kang et al. [62] introduced two additional primer sets into the original Bruce-ladder assay to improve the discrimination of *B. canis*, *B. ceti*, *B. microti*, and *B. inopinata* (Table S1, index A8). The first primer set targeting BMEIr02 (23S rRNA gene) was designed to produce a 344-bp amplicon specific only for *B. microti*. The second primer set, targeting the BMEII0722-0721 region, replaced the BMEI1436f-1435r primer pair of the original Bruce-ladder assay. According to these authors, *B. canis* and *B. abortus* have deletions in the *BMEII0722* gene of 12 bp and 94 bp, respectively, while *B. ceti* has an extended deletion of 972 bp within the *BMEII0721* gene. These deletions prevent amplicon production for *B. abortus* biovars 1- 6 and 9, strains RB5 and S19, *B. canis*, *B. ceti*, and *B. inopinata* to allow for differentiation of *B. canis* from *B. suis* strains and of *B. ceti* from *B. pinnipedialis* strains. This modified multiplex assay based on Bruce-ladder is a simple and rapid test capable of identifying 10 of 12 known *Brucella* species in a single test (Table S2, index A8).

All the above-mentioned assays employ PCR plus gel analysis. They have the intrinsic disadvantages of conventional PCR: a high risk of cross-contamination in manipulating amplicons for analysis, time intensity, and requirement for agarose gel preparation and electrophoresis compared to real-time assays.

## 4. Loop-Mediated Isothermal Amplification (LAMP)

Loop-mediated isothermal amplification (LAMP) was described by Notomi et al. [63] in 2000 as a novel method for fast and highly specific DNA amplification under isothermal conditions. The method utilizes a DNA polymerase and a set of four or six specific primers. The main feature of the method is application of the special primers (FIP and BIP) which contain sequences of both the sense and antisense strands of the target DNA. Initially, the forward inner primer (FIP) attaches to one strand of the template DNA and the DNA polymerase starts to synthesize the first amplicon (amplicon-1). Next, the forward outer primer (F3) initiates the synthesis of amplicon-2 on the same template DNA, which causes displacement of the earlier synthesized amplicon-1. The amplicon-1 becomes a template for the backward inner primer (BIP), which initiates synthesis of the amplicon-3. Then, the backward outer primer (B3) initiates synthesis of amplicon-4, which leads to displacement of the amplicon-3. The resulting amplicons form structures with two loops at each end, known as “dumbbell structures”. The loops contain loci complementary to FIP and BIP primers, which start a new cascade of synthesis events leading to the formation of several types of products. The cycling reaction results in about 10^9^ amplicons in less than one hour. The LAMP method is considered highly specific as it utilizes a set of four or six primers to recognize one target.

Ohtsuki et al. [64] developed two sets (P-1 and P-2) of six LAMP primers designed to detect the sequence of the *Brucella bcsp31* gene (Table S1, index A9). The assays were tested on 22 *Brucella* isolates of the core panel and 28 isolates of 18 non-*Brucella* species (Table 2, genera # 9, 10, 14, 28, 29, 41, 45, 60, 62, 64, 67, 68, and 70). In addition, the assays were used to analyze contaminated milk and infected organs from mice. The expected PCR product was found in all the *Brucella* strains tested (Table S2, index A9), but not in the representatives of non-*Brucella* species. The analytical limit of detection was 10 fg of genomic DNA.

Similar LAMP assays for the identification of *Brucella* genus or a single species were designed by various research groups [65,66,67,68,69]. Such assays proved to be simple and effective for the identification of *Brucella* in diagnostic samples and cultures. The assay may be conducted with simple equipment such as a water bath or with inexpensive constant-temperature instruments. The assay can incorporate fluorescent labels to enhance sensitivity and make direct reading possible. This methodology may be especially useful in resource limited settings where labs may lack adequate facilities, sophisticated equipment, and personnel trained in molecular biology.

## 5. Real-Time PCR

Real-time PCR, sometimes called quantitative polymerase chain reaction (qPCR, although qPCR is typically used to quantify rather than determine presence or absence), was an advancement from conventional PCR not requiring a post-amplification gel analysis. The method utilizes various DNA-intercalating or probe-attached fluorescent dyes (fluorophores) such as SYBR Green, fluorescein (FAM), cyanine (Cy3, Cy5, etc.), HEX, ROX, and many others, which make it possible to monitor the accumulation of PCR products directly during amplification. It is a rapid, sensitive, and specific technique. The simplest and most inexpensive variants of real-time PCR assays are based on the application of target sequence and incorporation of intercalating fluorophores (usually SYBR Green I) that increase in fluorescence intensity upon binding to double stranded DNA. The nonspecificity of intercalating dyes is the main disadvantage of their use in real-time PCR assays. Background fluorescence from the intercalation of nontarget DNA in the reaction tube can present a problem if high DNA concentrations are carried over from the test samples. Additionally, as the amplicon concentration increases and fluorescence intensity increases, fluorescence from primer dimer formation and amplification can provide a false signal. Melting curves of the amplicon products can discriminate between these primer dimer amplicons and target amplicons.

To increase the qPCR specificity, various fluorescently labeled oligonucleotide probes are used: TaqMan, fluorescence resonance energy transfer (FRET), minor groove binding (MGB), molecular beacons, and scorpion probes are examples of real-time PCR probes.

Redkar et al. [70] described three individual (uniplex) real-time PCR assays designed to detect the most pathogenic species of *Brucella*: *B. abortus*, *B. melitensis*, and *B. suis*. Each assay utilized two primers and two fluorescently labeled FRET probes (upstream and downstream) (Table S3, index A10). FRET is a phenomenon of energy transmission from one fluorescent molecule (donor) to another (acceptor) when the probes are in proximity, and when the emission spectrum of the donor overlaps with the absorption (excitation) spectrum of the acceptor. In the PCR assays described here, a pair of FRET probes represent such a donor–acceptor pair. The 3′ end of each upstream probe (FRET probe 1) is labeled with fluorescein (donor molecule), while the 5′ end of the downstream probe (FRET probe 2) is labeled with Cy5 dye (acceptor molecule). There are 1–4 bp separating the probes. As amplicons generated during PCR, FRET probes bind to them, allowing energy transfer from fluorescein to Cy5 and the emission of fluorescent light. This leads to an increase in the level of fluorescence, which is considered a positive result. The assays were tested on 20 reference *Brucella* strains (the core panel), 17 *Brucella* field isolates, and 7 non-*Brucella* strains (Table 2, genera # 6, 11, 45, 56, and 70). The developed assays identified and distinguished *B. abortus*, *B. melitensis*, and biovar 1 of *B. suis* (Table S2, index A10) but did not identify specific biovars. No cross-reaction with non-*Brucella* species was detected. The sensitivity of the assays, according to the authors, was 250 fg of DNA (~70 genome copies). In general, the assays proved to be rapid, reliable, and specific, but were nonreactive with biovars 2–5 of *B. suis*.

Newby et al. [71] evaluated three real-time PCR approaches designed for the field-portable Ruggedized Advanced Pathogen Identification Device (R.A.P.I.D., Idaho Technology, USA) utilizing a common primer pair, but different modes of detection of *Brucella abortus*: SYBR Green I, 5′-exonuclease TaqMan, and hybridization FRET (Table S3, index A11). The assays were tested on 19 *Brucella* strains (the core panel) and 8 non-*Brucella* strains (Table S2, genera # 11, 45, 56, and 63). The limit of detection (LOD) of all three assays was equal to 7.5 fg (~2 genome copies). The species specificity of the assays is given in Table S2, index A11. The SYBR Green I assay generated fluorescence in all *Brucella* and non-*Brucella* strains, thus showing a lack of specificity. The melting-curve analysis also failed to discriminate target from nontarget reactions. The 5′-exonuclease TaqMan assay identified all the target *B. abortus* strains as well as nontarget *B. canis* strains, suggesting *B. canis* and *B. abortus* share this locus. A better specificity was demonstrated by the hybridization FRET assay, which misidentified only one *B. canis* strain and only at high DNA concentration (greater than 10 pg). Since only two isolates of *B. canis* were tested, it is unclear whether the specificity of FRET probes is truly greater. Melting curve analysis of the FRET probes did not improve the assay. Probert et al. [72] redesigned the primers and probes for the genetic targets described by Redkar et al. [70] in order to create a multiplex TaqMan real-time PCR assay (Table S3, index A12). The resulting real-time PCR assay allowed rapid identification of *Brucella* spp., *B. abortus*, and *B. melitensis* in a single test. For *Brucella* spp. identification, the authors used primers and a probe targeting the *bcsp31* gene. The assay was tested on 61 *Brucella* strains (*B. abortus*, *B. melitensis*, *B. suis*, and *B. canis*) and an extended panel of 59 non-*Brucella* strains (Table 2, genera # 1, 2, 5, 6, 10, 11, 14, 17, 18, 25, 27–30, 33, 39, 43, 45, 46, 49, 53–55, 59, and 64–67, 70). All *Brucella* strains were reliably identified by the genus-specific primer/probe set (*bcsp31*) and the target species *B. abortus* and *B. melitensis* were successfully discriminated (Table S2, index A12). However, two *Brucella* strains phenotypically belonging to *B. abortus* were identified as *B. melitensis* using this assay. Sequence analysis of the omp2a locus suggests the two isolates represented atypical *Brucella* strains that share phenotypic and genotypic characteristics of both *B. abortus* and *B. melitensis*. This may represent differences in geographic strains as these two isolates were from an under-represented strain from the Middle East. The limit of detection for each set was 150 fg (~40 genome copies) of purified DNA. No significant fluorescence was registered for the non-*Brucella* strains. The advantage of the assay is its multiplex format that allows the identification of *Brucella* spp., *B. abortus*, and *B. melitensis* in a single test. Expansion of the *Brucella* panel to include all other *Brucella* species would be of interest and would determine the actual specificity of the primer sets. Bounaadja et al. [73] proposed three uniplex real-time PCR assays targeting genes *bscp31* and *per*, as well as the insertion sequence IS*711* for the detection of *Brucella* at the genus level (Tables S2 and S3, index A13). The assays were tested on 26 *Brucella* strains (the core panel, *B. ceti*, and *B. pinnipedialis*) and a large panel of 68 non-*Brucella* strains (Table 2, genera # 1, 4–6, 9, 11, 13, 15, 16, 20, 22, 23, 26–29, 34–36, 40, 49–51, 53, 56, 57, 60, 61, 63, 64, 66, 69, and 70). All assays demonstrated *Brucella* spp. specificity, while no cross-reactivity with non-*Brucella* strains was observed. Analytical sensitivity of the real-time PCR assays varied from 2 to 20 fg.

The effective performance of the described assays provides multiple options for the genus-specific identification of *Brucella* strains.

Hinić et al. [74] developed primers and TaqMan probes (Table S3, index A14) to perform seven individual reactions for the rapid detection of *Brucella* spp., and differentiation among the six classical *Brucella* species: *B. abortus*, *B. canis*, *B. melitensis*, *B. ovis*, *B. neotomae*, and *B. suis*. The assays were tested on 18 reference *Brucella* strains of the core panel, 47 *Brucella* field isolates, and 49 non-*Brucella* strains (Table 2, genera # 1, 3, 4, 6–8, 10, 11, 14, 15, 17, 20, 21, 24–27, 29–32, 34, 36, 37, 39, 40, 42, 44, 48, 49, 51, 53, 60, 62, 64–67, and 70). Seven pGEM^®^-T-Easy vector-based plasmids, containing the target sequences, were cloned and used by the authors in various concentrations to test the sensitivity of the real-time PCR assays. The limit of detection for the assays was about 10–100 plasmid copies. Each tested *Brucella* species had a unique profile (Table S2, index A14), with two exceptions. *B.* canis and *B. suis* biovar 4 both reacted with the TaqMan probes and shared the same agarose electrophoresis profile, while the *B. canis* field strain designated as D 368/94 had a positive PCR result but did not produce the expected 83 bp amplicon of PCR 4 characteristic for all *B. suis* and *B. canis* strains (data were not provided). All the non-*Brucella* species tested negative. These assays show promise for identification of *Brucella* species. Expansion of the test panel to include many more isolates of each species would increase confidence in the usefulness of these assays to identify the various species of *Brucella*. Foster et al. [75] developed species-specific real-time PCR assays targeting SNPs in six housekeeping and other genes: *abc*, *cysW*, *omp25*, *pip*, *rpoB*, and *trpE* (Table S3, index A15). The assays were tested on a diverse collection of 338 *Brucella* isolates (the core-panel and marine-mammal *Brucella* species) and 4 non-*Brucella* strains (Table 2, genus # 45). The assays were able to identify seven major *Brucella* species: *B. abortus*, *B. melitensis*, *B. canis*, *B. suis*/*B. canis*, *B. neotomae*, *B. ovis*, and *Brucella* spp. of marine mammals (Table S2, index A15). The reliable amplification for all assays started at 100 fg/μL. The DNA samples of non-*Brucella* strains (*Ochrobactrum anthropi*) were amplified in two assays (*abc*_205 and *rpoB*_2673) but only as a secondary (nonspecific) allele. These assays also show great promise for differentiation of *B. abortus* and *B. melitensis*, but more data are required to determine their value for discriminating the other *Brucella* species. Fretin et al. [76] proposed four locked nucleic acid (LNA)-based real-time PCR assays for the discrimination of *B. suis* strains at the biovar level using a set of critical SNP loci: *ptsP-1677*, *pyrH816–817*, *rpoB-244* and *dnaK-1005* (Table S3, index A16). LNA is a synthetic analog of DNA that exhibits high thermal stability. Even one mismatched LNA nucleotide can lead to a dramatic change in melting temperature. In these assays, four nucleotides of the TaqMan probes were substituted with LNA nucleotides. The resulting LNA real-time assays were tested on 137 field strains of *B. suis* and *B. canis*, as well as on the two control strains: *B. abortus* str. 544 and *B. melitensis* str. 16M. As a result, five different genotypes were identified corresponding to the five known biovars of *B. suis* (Table S2, index A16). However, the assays were not able to discriminate between *B. suis* bv. 4 and *B. canis.* Gopaul et al. [77] described seven species-specific and two genus-specific real-time PCR assays for differentiation of the six classical and two marine-mammal *Brucella* species. Each assay consisted of a pair of primers and two alternative minor groove binding (MGB) probes labeled with VIC or FAM fluorescent dye (Table S3, index A17). Each MGB probe in an assay was complementary to one of the two alleles of the corresponding SNP locus. A minor groove binding (MGB) probe is a short deoxyoligonucleotide labeled at 5′-end with a fluorophore and linked at 3′-end to a specific tripeptide, capable of binding to the minor groove of a DNA molecule. This increases the melting temperature and the specificity of the probe. The assays were validated using 303 *Brucella* isolates (the core panel, vaccine strains S19, RB51, and Rev.1, and marine-mammal *Brucella* species). Specificity of the assays was tested using five non-*Brucella* strains (Table 2, genus # 45). The allelic discrimination plots generated for each SNP assay showed a clear specific differentiation of the studied strains (Table S2, index A17). The limit of detection of each individual assay was 50 fg of DNA (~15 genomic copies). All five non-*Brucella* strains (*O. anthropi*) showed slightly positive results when tested by species-specific assays. However, application of the genus-specific assays based on the 16S rRNA sequence clearly proved these strains did not belong to the *Brucella* genus. Koylass et al. [78] reported the MGB assays designed by Gopaul et al. [77] more precisely identified *B. canis* strains than the original Bruce-ladder assay (García-Yoldi et al. [59]). These SNP assays also show excellent promise for the identification of *Brucella* species. The inclusion of an SNP for *B. microti* increases the value of the assay. In 2010, Gopaul et al. [79] reported five new MGB assays (Table S3, index A18), designed to identify, specifically, the vaccine strains using five SNP loci. Two assays targeting Rev.1 strain were tested using a panel of 277 *Brucella* isolates (predominantly *B. melitensis*), including 34 Rev.1 strains. Two assays targeting the S19 strain and one assay targeting RB51 were examined using a panel of 498 *Brucella* isolates (predominantly *B. abortus*). All the MGB assays clearly discriminated the vaccine and nonvaccine isolates (Table S2, index A18) and had limit of detection about 100 fg (~30 genomic copies). According to the authors, the MGB assays can be successfully used separately or in combination with other assays for identification of vaccine strains. Discrimination of vaccine strains from wild type strains is an important feature, since the veterinary vaccines in use are live-attenuated strains that can be isolated from vaccinated animals.

Winchell et al. [80] reported the development of seven individual real-time PCR assays designed to detect various genus-specific and species-specific *Brucella* markers (Table S3, index A19). Two of the target markers *Bspp* (specific for all *Brucella* spp.) and *Bsui* (specific for *B. suis*) represented unique genomic sequences, detectable using standard real-time PCR, while the other five (*Bcan*, *Bmar*, *Bmel*, *Bneo*, and *Boa*) contained SNP loci requiring a more sophisticated technique of allelic discrimination. For this purpose, the authors used an additional post-PCR step, called high-resolution melt (HRM) analysis, allowing differentiation between two amplicons differing by one or more nucleotides comparing their melting curves. The melting temperature of a DNA molecule depends on its nucleotide composition. The higher the GC content, the higher the temperature needed for DNA denaturation. During HRM analysis, the amplicons with intercalated SYBR Green I are heated gradually from 50°C to 95°C. When the temperature reaches the amplicon melting point, SYBR green I is released, and the fluorescence intensity is sharply reduced. Two amplicons differing by a single nucleotide will have a shift in melting curves (melting temperatures), allowing easy discrimination. It should be noted that not all SNPs can be readily detected by the HRM analysis, only those changing GC% of the fragment [81]. These assays were successfully tested on 153 *Brucella* strains (the core panel, vaccine strains, *B. ceti*, and *B. pinnipedialis*), as well as some clinical samples. The specificity of the assays was tested against a panel of 31 strains of closely related non-*Brucella* species (Table 2, genera # 5, 6, 28, 30, 41, 45, 46, 54, 56, 60, 67, and 70). The lower limit of detection for most markers was 100 fg. The *Bspp* marker detected all members of the *Brucella* genus. Other markers identified only the target species (Table S2, index A19). All the non-*Brucella* strains showed no cross-reactivity with the seven markers used. These assays represent a reliable allelic discrimination and real-time PCR approach for identifying *Brucella* species. A disadvantage of this assay is the inability to discriminate vaccine strains.

Gopaul et al. [82] designed an HRM multiplex (quadruplex) assay targeting five SNP markers allowing identification of five terrestrial *Brucella* species: *B. abortus*, *B. melitensis*, *B. ovis*, *B. suis*, and *B. canis* (Table S3, index A20). The assay was examined using 135 *Brucella* isolates (the core panel, marine mammal *Brucella* species, *B. inopinata*, *and B. microti*) and 23 non-*Brucella* strains (Table 2, genera # 45, 47, and 52). Each of the five target species demonstrated allelic discrimination and, thus, could be differentiated from other *Brucella* species (Table S2, index A20). In addition, according to the authors, the *B. microti* strain also has a unique SNP in a housekeeping gene. The limit of detection for the assay was about 100 fg DNA. No cross-reactivity with the non-*Brucella* strains was observed. In general, the quadruplex PCR assay of Gopaul et al. has an advantage over the previously described uniplex assays of Winchell et al., as it can identify the target species in a single reaction. These assays identify the six core species and the vaccine strains of *B. abortus*, and cluster the marine-mammal isolates in a single reaction.

Most recently, Girault et al. [83] introduced a 17-SNP-based high-resolution melting (HRM) assay to differentiate each *Brucella* spp.; *B. suis* biovars 1, 2, and 3; and the *B. melitensis* rev 1 vaccine strain. HRM is a post-PCR process and increases precision and accuracy for differentiating samples. This approach was tested against 1440 DNA samples of *Brucella* spp. from the European Reference Laboratory (EU-RL) and showed high discriminatory power. The process runs in hours and provides a new tool for species identification of *Brucella* samples. The methodologies are presented in this Special Issue with complete primer sets described in the paper’s Table 1 [83].

## 6. Restriction Fragment Length Polymorphism (RFLP)

Restriction fragment length polymorphism (RFLP) is a technique based on the standard PCR method with an additional step of amplicon treatment with restriction enzymes (restriction endonucleases and restrictase). DNA restriction sites occur throughout the genome, and occasionally polymorphisms such as SNPs, deletion, or insertions lead to the formation or loss of restriction sites. Digestion of amplicons or whole-genomic DNA preparations allows specific cleavage of the nucleic acid at the restriction site with subsequent resolution of the products using gel electrophoresis. The number and size of the resulting bands create profiles that are useful for the identification or genotyping of organisms.

Cloeckaert et al. [84] performed RFLP analysis of polymorphisms in genes *omp25*, *omp2a* and *omp2b*, encoding *Brucella* outer-membrane proteins (OMPs). They used three primer pairs and thirteen restriction enzymes (Table 3, index A21). The study was conducted on 77 strains and field isolates representing the 6 classical *Brucella* strains. Analysis of the *omp25* gene fragment digested by nine restriction enzymes revealed an absence of the *Eco*RV site in all *B. melitensis* strains and an ~50 bp deletion in all *B. ovis* strains. Analysis of the PCR products of the genes *omp2a* and *omp2b* digested by all the 13 restrictases revealed greater diversity, allowing discrimination between six *Brucella* species and even some biovars, except between *B. canis* and *B. suis* bv. 3 and 4 (Table S2, index A21).

Vizcaino et al. [85] carried out an analysis of the *omp31* gene of 73 *Brucella* strains (the core-panel species) using one primer pair and ten restriction enzymes (Table 3, index A22). Six enzymes (*Ban*I, *Hae*II, *Kpn*I, *Pvu*II, *Rsa*I, and *Sty*I) gave the same pattern for all the strains tested (data were not shown). Four other restriction endonucleases (*Ava*II, *Hae*III, *Sal*I, and *Sau*3AI) allowed classification of the *Brucella* strains into seven groups (including *B. abortus*, which did not produce any amplicons), that were hardly consistent with *Brucella* species (Table S2, index A22). However, *Sau*3AI was able to identify the *B. ovis* species, while enzyme *Ava*II can be useful for discrimination between *B. suis* bv.2, *B. canis* and all other strains, including *B. suis* bv.1, 3–5.

Clavareau et al. [87] reported an RFLP analysis of minke whale (*Balaenoptera acutorostrata*) B202R *Brucella* strain based on an IS*6501* probe and polymorphism of OMP genes. The study showed a unique profile of the B202R strain in comparison with the profiles of the six core *Brucella* species (data were not shown).

García-Yoldi et al. [86] used four primer pairs and a large number of restriction enzymes to analyze genes *omp22*, *omp25b*, *omp25c*, *omp25d*, and *omp31b*, encoding outer-membrane proteins (Table 3, index A23). The test was performed on 37 *Brucella* strains (the core-panel and marine-mammal *Brucella* species). The authors reported the absence of *Dde*I and a *Hinf*I restriction sites in the *omp25c*/*omp25d* genes of *B. abortus* biovar 6 and *B. ovis*, respectively. In addition, *B. ovis* strains have a 30 bp deletion close to the *omp22* gene. Among the studied genes, more polymorphisms were observed in the *omp31b*. RFLP analysis of this gene using a single enzyme *Dde*I allowed the classification of *Brucella* strains into four different groups: 1—*B. abortus*, 2—*B. melitensis*, 3—*B. ovis*, and 4—other species (Table S2, index A23). PCR-RFLP is quite time-consuming; in addition, the discriminatory power of the method is comparatively low.

## 7. Multilocus Sequence Analysis/Typing (MLSA/MLST)

Multilocus sequence analysis/typing (MLSA/MLST) is a technique that targets fragments of multiple conserved genes (historically, housekeeping genes) that contain various polymorphic sites. Based on the DNA sequence data for these targets, the studied organisms are assigned to designated sequence types (STs). The MLSA test is characterized by concatenation of the different target fragment sequences into a single sequence for further phylogenetic analysis.

Whatmore et al. [88] performed MLSA with nine primer sets (BruMLSA9) to examine polymorphisms in genes *gap*, *aroA*, *glk*, *dnaK*, *gyrB*, *trpE*, *cobQ*, *omp25* and *int-hyp* (Table 4, index A24). They tested 160 *Brucella* isolates (the core-panel and marine-mammal *Brucella* species) and identified 27 distinct STs (Table S2, index A24). The neighbor-joining tree constructed based on the STs classified *B. abortus*, *B. melitensis*, *B. ovis*, *B. neotomae*, and marine-mammal isolates into five relatively well-separated clusters, while *B. suis* biovars were widely scattered on the tree, placing *B. suis* bv. 3 and 4 and *B. canis* in one cluster. The BruMLSA-9 test in its original or modified form was successfully applied for *Brucella* typing by various research groups [89,90,91].

Whatmore et al. [92] extended the BruMLSA9 test described earlier with 12 additional housekeeping gene loci (Table 4, index A25). The new BruMLSA21 assay was applied to a collection of 508 isolates representing all 12 known *Brucella* species and resulted in 101 STs (Table S2, index A25). The BruMLSA21 assay confirmed the separation of *Brucella* strains into five main clades: *B. abortus*/*B. melitensis*, *B. suis*/*B. canis*, *B. ceti*/*B. pinnipedialis*, *B. ovis*/*B. papionis*, and *B. neotomae*.

## 8. Ligase Chain Reaction (LCR)

Ligase chain reaction (LCR) is a technique based on the use of a thermostable ligase for joining two adjacent oligonucleotide probes (upstream and downstream) tandemly bound to a genome locus containing a target SNP. The upstream probe is designed so that its 3′-end nucleotide is at the target SNP position. If the terminal nucleotide of the probe is complementary to the SNP locus nucleotide of the genomic DNA, the ligase concatenates it with the 5′-end nucleotide of the downstream probe to produce one long oligonucleotide fragment. This fragment can subsequently be amplified and detected using a real-time PCR thermal cycler.

Wattiau et al. [93] performed an LCR analysis targeting various SNPs in multiple housekeeping genes using Cy5-labeled universal forward primer, universal reverse primer and 16 padlock probes of different length (Table 5, index A26). A padlock probe (PLP) is a long oligonucleotide, whose ends are complementary to the target DNA, representing the upstream and downstream LCR probes, while the noncomplementary part forms a loop containing sites for specific primers. In case of successful PLP binding and matching, both its ends are joined by a ligase to form a circular DNA molecule, which serves as a matrix for the synthesis of the target amplicons. The assay was tested on 103 strains and field isolates representing 10 *Brucella* species known at that time, as well as 19 non-*Brucella* strains (Table 2, genera # 9, 14, 23, 28, 29, 45, 60, 61, 64, and 70). As a result, 27 capillary electrophoresis profiles were obtained allowing differentiation of *Brucella* at the species level, and only partially at the level of biovars (Table S2, index A26). No signal was detected from the non-*Brucella* strains. The assay discriminates vaccine strains but has a low sensitivity and is time-intensive.

## 9. Multiple Locus VNTR Analysis (MLVA)

Multiple locus VNTR analysis (MLVA) is a method widely used for bacterial genotyping. It assesses the number of repeats within multiple VNTR (variable-number tandem repeat) loci. The technique is quite like the standard PCR with endpoint product detection using gel or capillary electrophoresis. Various fluorescently labeled primers are used to provide amplicon sizing using capillary electrophoresis [94]. Now, MLVA genotypes can also be determined efficiently using in silico analysis of whole-genome sequences.

Bricker et al. [95] described a technique named “HOOF-Prints” (hypervariable octameric oligonucleotide fingerprints), targeted at the identification of the copy number of the octanucleotide DNA motif “AGGGCAGT” at eight loci in the genome. The authors used eight unique forward primers (labeled with HEX, FAM, and NED fluorescent dyes) and two types of reverse primers: Rev-1 and Rev-3 (Table S4, index A27). The PCR products were sized by electrophoresis on 3% Metaphor and 4% agarose gels, as well as by the high-throughput automated fluorescent DNA fragment analysis. The assay was tested on a panel of 19 pure cultures representing the six classical species, including vaccine strains as well as field strains. The authors revealed that none of the loci exhibited species- or biovar-specific alleles (Table S2, index A27). Isolates collected from different animals in the same herd had identical fingerprints. Whatmore et al. [96] reported a molecular-subtyping system based on 21 VNTR loci: the 8 earlier described HOOF-Prints loci and 13 novel VNTR markers (Table S4, index A28). The primer set “HOOF-Prints 7” was redesigned by the authors to improve its effectiveness. The sizes of the motifs (repeat units) within the new VNTR loci varied from 5 to 40 bp. The assay was tested on a collection of 121 *Brucella* isolates obtained worldwide and representing the 6 core *Brucella* species. According to the test, the *Brucella* isolates were divided into 119 genotypes. The analysis of genotypic clusters, with minor exceptions, corresponded to the conventional species designations. However, the authors decided that application of six of the novel loci (VNTR 7, 14, 21, 24, 26, and 27), characterized by longer tandem repeats, allowed the differentiation of all the *Brucella* isolates into 17 distinct genotypes (Table S2, index A28). Accordingly, the assay designed by Whatmore et al. was the most definitive method available at that time for *Brucella* genotyping.

Le Flèche et al. [97] evaluated 80 *Brucella* VNTR loci and selected a set of 15 optimal markers (Table S4, index A29) used for typing a larger collection of 257 *Brucella* strains including the core panel, as well as marine-mammal *Brucella* species. Nine of the markers were equivalent to those described earlier by Bricker et al. (TR2, TR6, and TR8) and Whatmore et al. (VNTR-2, 5a, 7, 17, 24, and 26), but the primers for their amplification were redesigned. This set of 15 VNTR markers was divided into two panels. Panel 1 comprised eight markers with a repeat length above 9 bp, while panel 2 included seven highly polymorphic octamers (repeat length was 8 bp). Both panels were tested on 21 typical *Brucella* strains (the core-panel and marine-mammal *Brucella* species) and 236 *Brucella* isolates. Panel 1 alone revealed 51 genotypes, but it was unable to distinguish *B. suis* biovar 4 and *B. canis*. Moreover, most *B. suis* biovar 3 strains shared the same genotype with *B. suis* biovar 1. Panel 2 identified 200 genotypes, but the resulting clustering was only approximately consistent with expected species and biovar assignments. The combination of both panels (MLVA-15) resulted in 204 genotypes grouped into several isolated clusters, supporting the current classification of the genus *Brucella* (Table S2, index A29). In 2007, Al Dahouk et al. [98] included an additional VNTR marker (bruce19) to panel 2 of the MLVA-15 assay and split the panel into two parts: panel 2A (VNTR loci 18, 19 and 21) and panel 2B (VNTR loci 4, 7, 9, 16 and 30), according to their diversity index. The MLVA-15/16 assay had the highest discriminatory power among the known PCR-based techniques for *Brucella* genotyping. Many research groups have successfully applied it for *Brucella* typing [99,100,101,102,103,104,105,106].

Huynh et al. [107] described another variant of the MLVA-15 assay (Table S4, index A30) composed of two multiplex PCRs. Forward primers were labeled with one of four 5′-fluorescent labels: 6-FAM, NED, PET, or VIC for amplicon discrimination by capillary electrophoresis. The assay was tested on 146 isolates and reference strains (including vaccine strains S19 and Rev.1) representing *B. abortus* bv. 1, 2, 4, 5, 6, and 9; *B. melitensis* bv. 1–3; *B. suis* bv. 1 and 4; *B. canis*; *B. ovis*; and *B. neotomae*. The MLVA system identified 136 genotypes and revealed unique species-specific alleles. A phylogenetic tree of the MLVA data constructed by the UPGMA method distributed the studied isolates into several main branches (Table S2, index A30).

In general, MLVA is a highly effective, robust, and reliable method of genotyping. Since sequencing methods are still quite expensive and time-consuming in much of the world, the MLVA techniques are an optimal alternative for the differentiation of *Brucella* strains, and the technique also provides phylogenetic analysis. Phylogenies provided using MLVA analysis are generally faster than being derived by genome sequence analysis including the whole-genome and core-genome SNP analysis, and generally provide greater resolution than MLST methods currently being employed. The open access database available for MLVA types allows researchers to download VNTR sets to compare new strains both phylogenetically and geographically (MLVA—Welcome to MicrobesGenotyping (https://microbesgenotyping.i2bc.paris-saclay.fr/) accessed 5 August 2022). 

## 10. Recommendations and Suggested Workflow

Here, we have focused on assays for discriminating species within the *Brucella* genus. Many of these molecular techniques are best applied to DNA extracted from known cultures. Confirmation of *Brucella* spp. from a diagnostic sample is beyond the scope of this review, but it is worth noting the importance of the process. It is important to recognize confirmation from a diagnostic sample should begin with a genus-level assay [108]. The techniques we have described here are best applied after confirmation of *Brucella* spp. Given the high risk of laboratory *Brucella* spp. infection, it is difficult and logistically challenging to always culture first and molecular type/confirm second. However, we do note there are many efforts underway to improve DNA isolation of the pathogen directly from a blood or tissue sample. The techniques listed here should be part of the solutions toward more rapid molecular-based identification.

This review reflects many of the approaches members of this author team have applied to *Brucella* diagnostics and typing throughout the former Soviet Union. The general approach we have used to molecular testing in our veterinary and human brucellosis surveillance efforts is a staged and hierarchical approach: (a) first, using a real-time PCR assay with a genus-specific target or targets (ideally a genus-specific but genus-wide target such as Tn711) for both genus-level detection and identification [109], followed by (b) an assay that can reliably distinguish species, at least for the species likely to be detected in the study (multiplexed SNP-based and/or conventional PCR assays [83,109]), followed by a genotyping tool that can provide something approaching strain-level differentiation (assays incorporating a range of VNTRs and/or more variable SNPs can potentially serve this purpose, although we have focused on MLVA). This approach can also be staged in the surveillance system being employed in an appropriate manner so that real-time detection/genus identification is carried out rapidly and at low cost in smaller labs/less-resourced labs in the system, with species identification being conducted at intermediate labs (or higher) in the system, and strain-level differentiation conducted at central laboratories that are better resourced. In the smaller labs collecting and processing surveillance samples, it is useful to be able to implement detection/genus identification and sometimes to also have a tool for species differentiation as well; when the latter is the case, a conventional PCR assay such as Bruce ladder may be more feasible than an SNP-based assay set, based on available resources. Strain-level differentiation in our studies has been performed on capillary sequencers because that was the diagnostic platform that was available, but under conditions in which it is possible to perform whole-genome sequencing on a next-generation platform, this would be ideal because in silico analysis of SNPs and MLVA will be possible with the same data, as long as the coverage and quality of the sequence data will allow reliable calls. Whole-genome sequencing would also allow for a range of other analyses to be carried out. In this staged/tiered approach, samples in which the genus is detected and identified/confirmed can be further analyzed to determine species (or confirm it if more extensive microbiological analysis has also been carried out), and, then, further analysis can be performed on either selected samples or all to differentiate strains and cluster them by SNP and/or MLVA approaches. In this approach, more conserved SNPs that identify a species or even a biovar can be used to provide information on the major branches in the phylogeny, more variable SNPs to provide lineages within those major branches, and, then, VNTRs (and/or whole-genome analysis) to differentiate further clusters and clonal lineages. Either whole-genome analysis or a tool such as MLVA is needed to identify recent disease transmission in a molecular epidemiologic analysis.

## 11. Conclusions

Because effective means for the vaccination and effective treatment of human brucellosis are lacking, veterinary disease control relying on the timely identification of infection within domestic livestock is essential. To date, there is no feasible way of monitoring disease in wild animals. Traditional methods of *Brucella* spp. identification depend on cultivation, biochemical analysis, and serological tests. Culture provides a source of DNA for use in molecular assays which can identify *Brucella* representatives quickly. Some assays will identify members in the genus, while others identify the specific species. The close genetic relatedness of previously established species within the genus *Brucella* has confounded the discrimination of some species, i.e., *B. canis* and *B. suis*. The recent discovery of additional *Brucella* species will require an additional survey of these isolates with existing assays to check for specificity. Conventional bacteriological, as well as some molecular, test methods put laboratory personnel at risk of infection, as they work with high titers of a pathogen that is highly infectious by aerosol. Polymerase chain reaction (PCR)-based techniques are reliable and effective for the identification of *Brucella* genera and perhaps even as a substitution for the biochemical and serology methods. There is now a wide variety of approaches based on PCR techniques: conventional PCR, real-time PCR, HRM analysis, loop-mediated isothermal amplification (LAMP), MLVA typing, and many others, but few have been through a rigorous validation incorporating blinded studies. The selection of an optimal approach with these methods is determined by the purpose of the study. Thus, the easiest way to rapidly reveal the presence of *Brucella* spp. in a culture sample is a conventional PCR, real-time PCR, or LAMP assay, targeting a genus-specific loci. Discrimination between several *Brucella* species can be effectively performed using multiplex or singleplex PCR methods, both conventional and real-time. Allelic discrimination assays show the best performance for discriminating species. The most reliable and cost-effective PCR-based method for the analysis of phylogenetic relationships of different *Brucella* strains is multiple-locus VNTR analysis (MLVA), while the most accurate and comprehensive but most expensive are whole-genome sequencing approaches (it should be noted that bioinformatics approaches are computationally intensive and need to clearly define the phylogenetic patterns searched for to discriminate strains in a MLVA-like approach). A major challenge in the attempt to move away from classical microbiological methods to only use DNA-based methods such as these is the frequent necessity to start with a pure isolate of the bacterium to extract a pure DNA sample for such analyses. It is difficult to do this without the microbiological skills required to obtain and properly process a pure isolate. PCR-based techniques validated for use with mixed cultures overcome some of this difficulty. Likewise, the use of molecular assays validated for use with clinical/environmental samples such as milk, urine and blood/blood culture, cheese, and other dairy products could speed up the identification of *Brucella* infection in these samples. Almost all the assays reviewed here were designed to detect the classical or core *Brucella* species: *B. abortus*, *B. canis*, *B. melitensis*, *B. neotomae*, *B. ovis*, and *B. suis*. Many were able to identify the two marine-mammal *Brucella* species: *B. ceti* and *B. pinnipedialis*. Some assays provided detection of the species *B. inopinata* and *B. microti*, while only one assay reported thus far detected the recently isolated atypical species *B. papionis* and *B. vulpis* [19]. The MLVA assays described should differentiate these atypical species into discreet clusters. In the current work, we reviewed reported molecular assays for *Brucella* assays using the data presented in the original publications. The use of real-time PCR, allelic discrimination assays, or MLVA genotyping assays appear to have the most discrimination power for the identification of *Brucella* species and, in some cases, biovars. It is yet to be determined how well biovars will cluster in genotyping assays. A direct comparison of these methods on the same blinded sample set would be an ideal way to compare their weaknesses and strengths. In lieu of such studies, selection and determination of the assay performance in a laboratory will provide a means of identification of *Brucella*. One should understand the limitations of the assays employed to prevent miscalls. Formal validation studies would expand the potential use of PCR-based *Brucella* assays while confirming the analytical characteristics as well as the diagnostic sensitivity and specificity. These data would provide confidence to users performing the assays.

## Figures and Tables

**Table 1:** The currently known Brucella species.

#	Species	Colony Phenotype	Authors and Year of Report	Biovar	Preferential Host(s)/Source	Human Pathogenicity
1	*B. melitensis*	Smooth	Hughes, 1893	1–3	Sheep, goat	High
2	*B. abortus*	Smooth	Bang, 1897	1–6, 9	Cattle	High
3	*B. suis*	Smooth	Traum, 1914	1, 3	Pig	High
				2	Wild boar, hare	Moderate
				4	Reindeer, caribou	High
				5	Rodent	None
4	*B. ovis*	Rough	Buddle, 1956	-	Sheep	None
5	*B. neotomae*	Smooth	Stoenner and Lackman, 1957	-	Desert wood rat	Moderate
6	*B. canis*	Rough	Carmichael and Bruner, 1968	-	Dog	Moderate
7	*B. ceti*	Smooth	Foster et al., 2007	-	Cetacean	Moderate
8	*B. pinnipedialis*	Smooth	Foster et al., 2007	-	Seal	Moderate
9	*B. microti*	Smooth	Sholtz et al., 2008	-	Vole, fox, soil	No data
10	*B. inopinata*	Smooth	Sholtz et al., 2010	-	Human	Moderate
11	*B. papionis*	Smooth	Whatmore et al., 2014	-	Baboon	No data
12	*B. vulpis*	Smooth	Sholtz et al., 2016	-	Red fox	No data

**Table 2:** Bacterial genera used for specificity test in the reviewed assays.

#	Genus	#	Genus	#	Genus	#	Genus	#	Genus
1	*Acinetobacter*	15	*Campylobacter*	29	*Francisella*	43	*Neisseria*	57	*Rhodococcus*
2	*Actinobacillus*	16	*Candida*	30	*Haemophilus*	44	*Nicoletella*	58	*Rhodospirillum*
3	*Actinomyces*	17	*Capnocytophaga*	31	*Helicobacter*	45	*Ochrobactrum*	59	*Roseomonas*
4	*Aeromonas*	18	*Cardiobacterium*	32	*Histophilus*	46	*Oligella*	60	*Salmonella*
5	*Afipia*	19	*Chlamydia*	33	*Kingella*	47	*Paenochrobactrum*	61	*Serratia*
6	*Agrobacterium*	20	*Citrobacter*	34	*Klebsiella*	48	*Paracoccus*	62	*Shigella*
7	*Arcanobacterium*	21	*Clostridium*	35	*Lactobacillus*	49	*Pasteurella*	63	*Sinorhizobium*
8	*Arcobacter*	22	*Corynebacterium*	36	*Listeria*	50	*Phyllobacterium*	64	*Staphylococcus*
9	*Bacillus*	23	*Coxiella*	37	*Mannheimia*	51	*Proteus*	65	*Stenotrophomonas*
10	*Bartonella*	24	*Edwardsiella*	38	*Mesorhizobium*	52	*Pseudochrobactrum*	66	*Streptococcus*
11	*Bordetella*	25	*Eikenella*	39	*Moraxella*	53	*Pseudomonas*	67	*Vibrio*
12	*Bradyrhizobium*	26	*Enterobacter*	40	*Mycobacterium*	54	*Psychrobacter*	68	*Wolbachia*
13	*Brevibacillus*	27	*Enterococcus*	41	*Mycoplana*	55	*Ralstonia*	69	*Xanthomonas*
14	*Burkholderia*	28	*Escherichia*	42	*Mycoplasma*	56	*Rhizobium*	70	*Yersinia*

**Table 3:** Primers from the reviewed PCR-RFLP assays.

Index	Assays, Authors	Primer/Probe	Sequence (5′-3′)	Target	Encoded Product	Restriction Enzymes Used
A21	PCR-RFLP, Cloeckaert et al. [84]	25A	GGACCGCGCAAAACGTAATT	*omp25*	Outer membrane proteins	*AluI*, *BanI*, *BglII*, *ClaI*, *EcoRI*, *HaeIII*, *HinfI*, *KpnI*, *NcoI*, *PstI*, *PvuII*, *StyI*, *TaqI*
		25B	ACCGGATGCCTGAAATCCTT			
		2aA	GGCTATTCAAAATTCTGGCG	*omp2a*		
		2aB	ATCGATTCTCACGCTTTCGT			
		2bA	CCTTCAGCCAAATCAGAATG	*omp2b*		
		2bB	GGTCAGCATAAAAAGCAAGC			
A22	PCR-RFLP, Vizcaino et al. [85]	31st	TGACAGACTTTTTCGCCGAA	*omp31*	31 kDa Outer-membrane proteins	*AvaII*, *BanI*, *HaeII*, *HaeIII*, *KpnI*, *PvuII*, *RsaI*, *SalI*, *Sau3AI*, *StyI*
		31ter	CATTCAGGACAATTCCCGCC			
A23	PCR-RFLP, García-Yoldi et al. [86]	22F	CGCGCTGATATCGACATGAC	*omp22*	Outer-membrane proteins	*DdeI*, *Hpy188I*, *DdeI*, *HinfI*, *DdeI*, *BsmI*, *HinfI*, *EcoRV*, *and other enzymes*
		22R	CCCGGCTGTTACATATGCTG			
		25cdF	CCGCCTGCTGTGTCCTGTTT	*omp25cd*		
		25cdR	GGCCGCGAAATAGACCAGAA			
		25bF1	CGGGCCGCTTTTTTACTGTT	*omp25b*		
		25bR1	GTGCGCCGCCGTTCTAATTC			
		31bF	CGTCGCCTTCCTGTCATC	*omp31b*		
		31bR	GCCGCAGTTCAATGATGT			

**Table 4:** Primers from the reviewed MLSA assays.

Assays, Authors	Primer Pair	Oligo Type	Sequence (5′-3′)	Gene/Locus	Encoded Product	Amplicon Size
MLSA-9 assay, Whatmore et al. [88]	1	F-primer	YGCCAAGCGCGTCATCGT	*gap*	*Glyceraldehydes 3-phosphate dehydrogenase*	589 bp
		R-primer	GCGGYTGGAGAAGCCCCA			
	2	F-primer	GACCATCGACGTGCCGGG	*aroA*	*3-phosphoshikimate 1-carboxyvinyltransferase*	565 bp
		R-primer	YCATCAKGCCCATGAATTC			
	3	F-primer	TATGGAAMAGATCGGCGG	*glk*	*Glucokinase*	475 bp
		R-primer	GGGCCTTGTCCTCGAAGG			
	4	F-primer	CGTCTGGTCGAATATCTGG	*dnaK*	*Chaperone protein*	470 bp
		R-primer	GCGTTTCAATGCCGAGCGA			
	5	F-primer	ATGATTTCATCCGATCAGGT	*gyrB*	*DNA gyrase B subunit*	469 bp
		R-primer	CTGTGCCGTTGCATTGTC			
	6	F-primer	GCGCGCMTGGTATGGCG	*trpE*	*Anthranilate synthase*	486 bp
		R-primer	CKCSCCGCCATAGGCTTC			
	7	F-primer	GCGGGTTTCAAATGCTTGGA	*cobQ*	*Cobyric acid synthase*	422 bp
		R-primer	GGCGTCAATCATGCCAGC			
	8	F-primer	ATGCGCACTCTTAAGTCTC	*omp25*	*25 kDa outer-membrane protein*	490 bp
		R-primer	GCCSAGGATGTTGTCCGT			
	9	F-primer	CAACTACTCTGTTGACCCGA	*int-* *hyp*	*Upstream and extreme 5′ of hypothetical protein (BruAb1_1395)*	430 bp
		R-primer	GCAGCATCATAGCGACGGA			
MLSA-21 assay, Whatmore et al. [92] (only 12 new primers are shown)	10	F-primer	GGTGCTGTTCACGCTGGAA	*prpE*	*Propionate-CoA ligase*	468 bp
		R-primer	AGGTTTTCGCAGGCGGCGAA			
	11	F-primer	TGTGTTCGGCAAGCCTTTG	*caiA*	*Acyl-CoA dehydrogenase*	449 bp
		R-primer	GGTCAAAAGACGTGCCACA			
	12	F-primer	CGTCACTTCCTGGATCATTTC	*csdB*	*Cysteine desulfhydrase*	487 bp
		R-primer	GCCACCGACGCTTATGAGAA			
	13	F-primer	CCTCGTAAAGCGCCTTCC	*soxA*	*Sarcosine oxidase alpha subunit*	486 bp
		R-primer	TGTTCGATGCCTCCACATTGG			
	14	F-primer	TCAACCGGATGAAGGAAGTC	*leuA*	*2-isopropylmalate synthase*	482 bp
		R-primer	CCCTCGATAGTCTTGGTGACA			
	15	F-primer	ATCGCCCGTTCGGTGAC	*mviM*	*Glucose-fructose oxidoreductase precursor*	447 bp (size variants identified at these loci)
		R-primer	TGTTCGCCGTCCTTGTCC			
	16	F-primer	CGACCATGTCAATATGAGCC	*fumC*	*Fumarate hydratase C*	452 bp
		R-primer	GATATCGTTGGCGATCTTGAA			
	17	F-primer	CGTGAAATAACCTGATCTCAC	*fbaA*	*Fructose-bisphosphate aldolase*	458 bp
		R-primer	CATGCCGGTTTCAAGCGAAC			
	18	F-primer	TTTCAGTGCGCTCGAACAG	*ddlA*	*D-alanine-D-alanine ligase A*	553 bp
		R-primer	GTTCTTCAATGATGAGATTAAA			
	19	F-primer	GTGGGCGTGCAGCCTTTCG	*putA*	*Proline dehydrogenase*	527 bp
		R-primer	CCTGTGTGAGTACGAGCGG			
	20	F-primer	ACATCCAAGCTGACCGAC	*mutL*	*DNA mismatch repair protein*	549 bp
		R-primer	TCCCGTGCGATCACATCCGA			
	21	F-primer	GAAGGCCGCATCCCACTG	*acnA*	*Aconitate hydratase*	490 bp
		R-primer	GCGGCGAGGCAAGGTAAT			

**Table 5:** Primers and padlock probes (PLP) from the reviewed LCR assay.

Index	Assays, Authors	Primer/Probe	Sequence (5′-3′)	Target	Encoded Product	Amplicon Size
A26	LCR assay, Wattiau et al. [93]	UR ^a^	GACGATGAGTCCTGAGTAA	*—*	N/A	*—*
		UF ^b^	CCGAGATGTACCGCTATCGT	*—*	N/A	*—*
		cUR ^c^	TTACTCAGGACTCATCCTC	*—*	N/A	*—*
		PLP-A	GCCGACAAGATCACGCCCA-cUR-AA-UF-CTGGGCATCTGCGCG	*glk-1403G*	glucokinase	73 bp
		PLP-B	GACACGCCCTTCGATGCGT-cUR-AA-UF-AGAATTTGCTCGCCGGC	*glk-1344G*	glucokinase	75 bp
		PLP-C	CCAGACGGGCGCCAAG-cUR-AA-UF-CATACGCTTGCCAATTATTTCCA	*trpE-2858A*	anthranilate synthase	78 bp
		PLP-D	TAGCCAAGGTAAAGACCGGTATAGCC-cUR-AA-UF-GGCCTTGTTCCAGCCA	*omp25-3627A*	25 kDa outer-membrane protein	81 bp
		PLP-E	AAGCCTCGCTGGATATTGATGGC-cUR-TT-UF-ATTATCTGGCTGAAGGGCTGA	*cobQ-3445A*	cobyric acid synthase	83 bp
		PLP-F	GCGGCGTTTATCTTTCGGGTAGCTA-cUR-AA-UF-GCTCATTTTCATGGCGCATA	*glk-1557A*	glucokinase	84 bp
		PLP-G	ACCCGCACCGGCCTG-cUR-AA-UF-TGATACTACTATGCAATGTGCTGATGAACCCA	*aroA-677A*	3-phosphoshikimate 1-carboxyvinyltransferase	86 bp
		PLP-H	CATCGACCTGAAGAACGACAAGC-cUR-TT-UF-TATCCGAGTTCAAGAAGGAAAGTGA	*dnaK-1654A*	chaperone protein	87 bp
		PLP-I	GCCTTCAATAGCGCGCGC-cUR-AA-UF-CGTCGCGTTAGACAGCTCATGGCCACCCGCC	*ptsP-1677G*	phosphoenolpyruvate-protein phosphotransferase	88 bp
		PLP-J	CACCAGCGGGCCGGA-cUR-AA-UF-TAGTCACATATCATGCTATGAAATCCACATCGGGCA	*cobQ-3224A*	cobyric acid synthase	90 bp
		PLP-K	TTCTCGATCGCGGGC-cUR-AA-UF-GGTTCGCTTACGTTGCATAGTGCTCACCCACAAGGAAG	*pyrH-817G*	uridylate kinase	92 bp
		PLP-L	ACCAGAACCACTTCGTCAATTTCG-cUR-AA-UF-ATCCGGTCTCATCGCTGAATGGTCATGCCGCCA	*dnaK-1928T*	chaperone protein	96 bp
		PLP-M	GTTTCGATCCTGCTGGTCGATCA-cUR-T-UF-ATGGTCGCCTATACTTATATCAAAGGTGGCTGAGGGA	*trpE-2796A*	anthranilate synthase	99 bp
		PLP-N	CTGGAAGTTCCAGCCAGCAAACG-cUR-AA-UF-CGATCCGATTACAGGCCGATCCGTATACGATCTGGTCCTT	*omp25-3715A*	25 kDa outer-membrane protein	102 bp
		PLP-O	ACTGTCCGCAAGCTTCAAGC-cUR-TT-UF-AAAATTTAACGTTCCTAAAGCTGAGTCTGCCCGGCCATTATGGTG	*IS711*	transposasa	104 bp
		PLP-P	ATGAATGCCGTCAGCGCG-cUR-TT-UF-ATTTGACGAACGTATGCCGCTTAACTCAAATCATCCACCGAAGTTGGATGTTA	*rpoB-265A*	DNA-directed RNA polymerase beta chain	110 bp

^a^ Universal reverse primer. ^b^ Universal forward primer (5′ Cy5-labeled). ^c^ Sequence complementary to the universal reverse primer. N/A = not available.

## Data Availability

Not applicable.

## Supplementary Materials

The following supporting information can be downloaded at: https://www.mdpi.com/article/10.3390/microorganisms10081584/s1. Table S1. Primers from the reviewed PCR and LAMP assays. Table S2. Discriminatory power of the reviewed assays. Table S3. Primers and probes from the reviewed real-time PCR assays. Table S4. Primers from the reviewed MLVA assays.
